# Reaching Agreement in Quantum Hybrid Networks

**DOI:** 10.1038/s41598-017-05158-7

**Published:** 2017-07-20

**Authors:** Guodong Shi, Bo Li, Zibo Miao, Peter M. Dower, Matthew R. James

**Affiliations:** 1 Research School of Engineering, Australian National University, Canberra, ACT 0200 Australia; 2 Key Lab of Mathematics Mechanization, Chinese Academy of Sciences, Beijing, 100190 China; 3 Department of Electrical & Electronic Engineering, The University of Melbourne, Parkville, Victoria, 3010 Australia

## Abstract

We consider a basic quantum hybrid network model consisting of a number of nodes each holding a qubit, for which the aim is to drive the network to a consensus in the sense that all qubits reach a common state. Projective measurements are applied serving as control means, and the measurement results are exchanged among the nodes via classical communication channels. In this way the quantum-opeartion/classical-communication nature of hybrid quantum networks is captured, although coherent states and joint operations are not taken into consideration in order to facilitate a clear and explicit analysis. We show how to carry out centralized optimal path planning for this network with all-to-all classical communications, in which case the problem becomes a stochastic optimal control problem with a continuous action space. To overcome the computation and communication obstacles facing the centralized solutions, we also develop a distributed Pairwise Qubit Projection (PQP) algorithm, where pairs of nodes meet at a given time and respectively perform measurements at their geometric average. We show that the qubit states are driven to a consensus almost surely along the proposed PQP algorithm, and that the expected qubit density operators converge to the average of the network’s initial values.

## Introduction

Consensus seeking over complex networks has played a foundational role in the development of distributed computation and networked control systems^1, 2^. How a set of isolated processors communicating only by means of two-party messages reach a common state in the presence of faulty nodes was a prior concern for fault-tolerant distributed computation^3^. Distributed controller design that drives a network of autonomous agents to certain consensus state such as the network average or some leader’s state^4^ turned out to be a primary step towards control, estimation, and optimization of networked control systems^1^. In the past decades, tremendous research efforts have been devoted to efficient design and convergence analysis of consensus and synchronization algorithms motivated by various social, engineering, and physical systems^5–9^.

In particular, consensus over quantum networks where node states are in quantum space and algorithms must be implemented by feasible quantum means has drawn attention^10, 11^. Quantum particles (subsystems) can be interconnected by local environments which are by themselves also quantum systems, the resulting state evolution will lead to a symmetric state consensus over such a quantum network, a concept introduced in ref. 10. The reduced states of the nodes will in turn asymptotically tend to the average of the nodes’ initial reduced states, in the almost sure sense along the discrete algorithm^10^ and deterministically along a quantum consensus master equation^11^. Such methods are essentially coherent quantum control for open quantum systems^12^, where the involved local environments can only be engineered at a small scale. On the other hand, many types of quantum networks, especially quantum communication networks, are hybrid in the sense that both quantum and classical parts co-exist^13, 14^. Quantum operations (often being measurements) can be performed locally and then the outcomes of the measurements are exchanged via classical communications, leading to the so-called local-operation classical-communication (LOCC) networks which have served as protocols for quantum cryptography or potential tools for engineering complex quantum states^15^. Measurement-based quantum control has also been demonstrated as effective means of manipulating quantum states both theoretically and experimentally^16–19^.

In this paper, we consider a consensus seeking problem over a quantum hybrid network consisting of a number of nodes each holding a qubit, where projective measurements are applied and the measurement results are exchanged. This theoretical simplification has neglected the effects of coherent states and joint operations in realistic quantum information processing networks, but the quantum-operation/classical-communication nature of LOCC networks has been preserved and highlighted. The problem of centralized optimal path planning for the network with all-to-all classical communications is shown to be a stochastic optimal control problem, whose computation and communication complexities are analyzed. We also develop a distributed Pairwise Qubit Projection (PQP) algorithm, where pairs of nodes meet at a given time and respectively perform measurements at their geometric average. The qubit states are driven to a consensus almost surely along the proposed PQP algorithm. The expected qubit density operators actually converge to the average of the network’s initial values, consistent with the work^10, 11^ for open quantum networks. Some preliminary results of the current work were announced at Australian Control Conference in 2016^20^.

## Results

### A Simplified Hybrid Quantum Network Model

Let a network of nodes be indexed in the set ${\rm{V}}=\{1,\ldots ,N\}$. Each node holds a qubit, i.e., a quantum system whose state space ${\mathcal H}$ is a two-dimensional Hilbert space. Let |0〉 and |1〉 form an orthogonal basis of the qubit space ${\mathcal H}$. Projective measurements can be performed at the individual qubits, respectively. An available projective measurement M_*α*_ is described by its two eigenstates

$$\cos \,\alpha |0\rangle +\,\sin \,\alpha |1\rangle ,$$

and

$$\cos \,(\alpha +\pi \mathrm{/2)}|0\rangle +\,\sin \,(\alpha +\pi \mathrm{/2)}|1\rangle .$$

We assume that the measurements are in the set

$$ {\mathcal M} =\{{{\rm{M}}}_{\alpha }:\alpha \in [0,\pi \mathrm{/2})\}.$$

The outcomes of a measurement M_*α*_ are indexed by $\ltimes$, corresponding to eigenstate $\cos \,\alpha |0\rangle +\,\sin \,\alpha |1\rangle$, and $\rtimes$, corresponding to eigenstate $\cos (\alpha +\pi \mathrm{/2)}|0\rangle +\,\sin (\alpha +\pi \mathrm{/2)}|1\rangle$. For the ease of presentation we will sometimes identify a measurement in the set ${\mathcal M}$ with its angle $\alpha \in [0,\pi \mathrm{/2)}$ since there is a natural one-to-one correspondence between the elements in ${\mathcal M}$ and angles in the interval [0, *π*/2).

Time is slotted for $t=0,1,\ldots$. The state space of the qubits ${\mathscr{S}}\subseteq  {\mathcal H}$ contains all possible outcomes of the measurements:

$${\mathscr{S}}=\{\,\cos \,\alpha |0\rangle +\,\sin \,\alpha |1\rangle :\alpha \in [0,\pi )\}.$$

The state of the qubit held by node *i* (or simply, qubit *i*) at time *t* is denoted by ${{\bf{x}}}_{i}(t)\in {\mathscr{S}}$. Similarly, noting that there is a one-to-one correspondence between a state in ${\mathscr{S}}$ and an angle in [0, *π*), we will identify **x**_*i*_(*t*) with its angle whenever convenient (This is to say, **x**_*i*_(*t*) can simply represent an angle ${{\bf{x}}}_{i}(t)\in [0,\pi )$, or represents a vector $\cos ({{\bf{x}}}_{i}(t))|0\rangle +\,\sin ({{\bf{x}}}_{i}(t))|1\rangle$). The network of nodes is interconnected by classical communications. At each time *t*, node *i* performs a measurement, denoted **u**_*i*_(*t*) and selected in the set ${\mathcal M}$, whose outcomes can be exchanged via the classical communication links. The goal is to design efficient rules for the selection of the **u**_*i*_(*t*), so that the **x**_*i*_(*t*) will tend to a common state.

**Figure 1:**
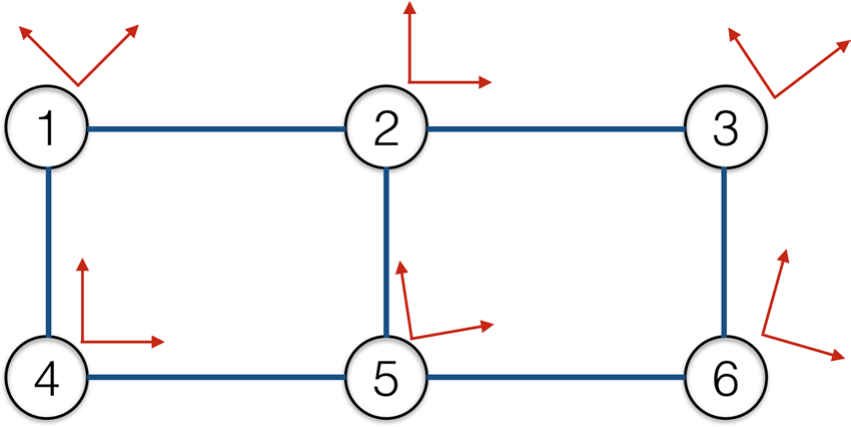
An illustration of a six-node quantum hybrid network: There is a qubit at each node, respectively; Projective measurements are performed at the individual qubits; Nodes are interconnected by classical communication links so that the outcomes of the measurements can be exchanged.

We give an example of the considered hybrid quantum network with 6 nodes in Fig. 1. Such a model is obviously a theoretical simplification since two important elements in real-world quantum communication or hybrid quantum information processing, coherent quantum states and joint measurements over multiple qubits, have been neglected^15^. However, despite of its simplicity, the model still captures the most fundamental aspect of local-operation classical-communication (LOCC) networks, where random outcomes of local quantum operations are shared with classical communications. More importantly, as will be seen from the results and the analysis, although coherent quantum states and joint measurements will change the state space, feasible actions, and state transition probabilities, the nature of the problem remains the same. Therefore, we would like to focus on this simple model in the current work where neat and analytical results become available with great clarity.

### Centralized Solution

In this section, we investigate the scenario when the nodes are equipped with all-to-all classical communications and derive the optimal rules for measurement sequence selections at the qubits.

#### Finite Horizon Optimal Policy

We stack the states of the qubits into an *N* dimensional column vector by ${\bf{x}}(t)={({{\bf{x}}}_{1}(t)\ldots {{\bf{x}}}_{N}(t))}^{{\rm{{\rm T}}}}$. The vector ${\bf{u}}(t)={({{\bf{u}}}_{1}(t)\ldots {{\bf{u}}}_{N}(t))}^{{\rm{{\rm T}}}}$ denotes the selection of measurements performed. The outcome of the measurement *u*_*i*_(*t*) is ${{\bf{y}}}_{i}(t)\in \{ \ltimes ,\rtimes \}$. We also denote ${\bf{y}}(t)={({{\bf{y}}}_{1}(t)\ldots {{\bf{y}}}_{N}(t))}^{{\rm{{\rm T}}}}$. Suppose the process ends at *t* = *T* for some integer *T* ≥ 1. The measurement selection decision is denoted by

$$\sigma ={\sigma }_{0}\times {\sigma }_{1}\cdots \times {\sigma }_{T-1}$$

where the *σ*_*s*_ assigning the value of **u**(*s*). The decision *σ*_*s*_ can depend on all information available by the time slot $s\in \{0,1,\ldots ,T-1\}$: **x**(*t*), **y**(*t*) for $t=0,\ldots ,s$ and **u**(*t*) for $t=0,\ldots ,s-1$. Formally we have

$${\bf{u}}(s)={\sigma }_{s}({\bf{x}}(t),{\bf{y}}(t),t=0,\ldots ,s;\,{\bf{u}}(t),t=0,\ldots ,s-1)$$

with *σ*_*s*_(·) can be an arbitrary function that takes values in ${ {\mathcal M} }^{N}$ for $s=0,1,\ldots ,T-1$. All such decisions *σ* are put in a set ${\Upsilon }_{T}$.

For any fixed measurement decision *σ*, the agreement displacement at time *T* is characterized by the expected network fidelity:

$${f}_{\sigma }(T)={{\mathbb{E}}}_{\sigma }\sum _{i,j=1}^{N}\,|\langle {{\bf{x}}}_{i}(T)|{{\bf{x}}}_{j}(T)\rangle |$$

where ${{\mathbb{E}}}_{\sigma }$ captures all randomness generated by the quantum measurements as well as possible random measurement decisions. The evolution of node states is governed by the quantum measurement principles and can be written as

$${\mathbb{P}}({\bf{y}}(t)={({y}_{1}\ldots {y}_{N})}^{{\rm{{\rm T}}}}|{\bf{x}}(t),{\bf{u}}(t))=\prod _{i}\,{|\langle {{\bf{x}}}_{i}(t)|{{\bf{u}}}_{i}^{{y}_{i}}(t)\rangle |}^{2}$$

where ${{\bf{u}}}_{i}^{{y}_{i}}(t)=\,\cos ({{\bf{u}}}_{i}(t))|0\rangle +\,\sin ({{\bf{u}}}_{i}(t))|1\rangle$ for ${y}_{i}= \ltimes$ and ${{\bf{u}}}_{i}^{{y}_{i}}(t)=\,\cos ({{\bf{u}}}_{i}(t)+\pi \mathrm{/2)}|0\rangle +\,\sin ({{\bf{u}}}_{i}(t)+\pi \mathrm{/2)}|1\rangle$ for ${y}_{i}=\rtimes$. By plain calculation we can further write

$${\mathbb{P}}({\bf{y}}(t)={({y}_{1}\ldots {y}_{N})}^{{\rm{{\rm T}}}}|{\bf{x}}(t),{\bf{u}}(t))=\prod _{i}\,{\cos }^{2}\,({{\bf{u}}}_{i}^{{y}_{i}}(t)-{{\bf{x}}}_{i}(t)).$$

Here we have identified ${{\bf{u}}}_{i}^{{y}_{i}}(t)$ and **x**_*i*_(*t*) with their angles. Note that, the value and distribution of **x**(*t* + 1) is fully determined by **y**(*t*) and **u**(*t*). This is to say **x**(*t*) is Markovian. Finding the policy *σ* that miximizes *f*_*σ*_(*T*) is a stochastic optimal control problem^21^.

The optimal policy σ* that maximizes *f*_*σ*_(*T*) can be obtained as follows. Clearly σ* is Markovian in the sense that **u**(*t*) depends only on **x**(*t*) for all $t=\mathrm{0,}\ldots ,T-1$ in the decision profile σ*. Introduce $u={({u}_{1}\ldots {u}_{N})}^{{\rm{{\rm T}}}}\in {{\mathscr{S}}}^{N}$ and $y={({y}_{1}\ldots {y}_{N})}^{{\rm{{\rm T}}}}\in {\{ \ltimes ,\rtimes \}}^{N}$. Define a function ${\bf{Q}}(u,y)={({q}_{1}\ldots {q}_{N})}^{{\rm{{\rm T}}}}\in {{\mathscr{S}}}^{N}$ by *q*_*i*_ = *u*_*i*_ if ${y}_{i}= \ltimes$ and *q*_*i*_ = *u*_*i*_ + *π*/2 if ${y}_{i}=\rtimes$. Introduce the cost-to-go function $C(\cdot ,\cdot ):{S}^{N}\times \{0,1,\ldots ,T\}\to {\mathbb{R}}$ defined by

$$C(x,t)=\mathop{{\rm{\max }}}\limits_{\sigma \in {\Upsilon }_{T}}\,{{\mathbb{E}}}_{\sigma }\,(\sum _{i,j=1}^{N}\,|\langle {{\bf{x}}}_{i}(T)|{{\bf{x}}}_{j}(T)\rangle ||{\bf{x}}(t)=x).$$

Then by a standard dynamic programming argument there holds

1 $$C(x,t)=\mathop{{\rm{\max }}}\limits_{u\in { {\mathcal M} }^{N}}\,\sum _{y\in {\{ \ltimes ,\rtimes \}}^{N}}\,{\mathbb{P}}({\bf{y}}(t)=y|{\bf{x}}(t)=x,{\bf{u}}(t)=u)\cdot C({\bf{Q}}(u,y),t+\mathrm{1)}$$

for $x\in {{\mathscr{S}}}^{N}$ and $t=\mathrm{0,1},\ldots ,T-1$. The boundary condition of (1) is

$$C(x,T)=\sum _{i,j=1}^{N}\,|\langle {x}_{i}|{x}_{j}\rangle |,\,x={({x}_{1},\ldots ,{x}_{n})}^{{\rm{{\rm T}}}}\in {{\mathscr{S}}}^{N}.$$

The optimal decision σ* is given by

2 $${\sigma }_{t}^{\ast }({\bf{x}}(t))={\rm{\arg }}\,\mathop{{\rm{\max }}}\limits_{u\in { {\mathcal M} }^{N}}\,\sum _{y\in {\{ \ltimes ,\rtimes \}}^{N}}\,{\mathbb{P}}({\bf{y}}(t)=y|{\bf{x}}(t),{\bf{u}}(t)=u)\cdot C({\bf{Q}}(u,y),t+\mathrm{1)}$$

for $$t=0,\ldots ,T-1$$.

#### Infinite Horizon Optimal Policy

Next, we consider an infinite horizon scenario when the optimality criteria is given by the minimal steps in expectation required for reaching a perfect agreement in the network. Let

$$\sigma ={\sigma }_{0}\times {\sigma }_{1}\times \cdots $$

be a measurement selection policy for the entire time horizon, where for any $s=0,1,\ldots ,{\sigma }_{s}$ maps to ${ {\mathcal M} }^{N}$ from all available information up to time *s*. All such decisions are put in the set ${\Upsilon }_{\infty }$. Consider the expected number of steps of reaching agreement at the qubits:

$${g}_{\sigma }={{\mathbb{E}}}_{\sigma }(\mathop{{\rm{\inf }}}\limits_{t}\{t\ge 0:\,{{\bf{x}}}_{1}(t)=\cdots ={{\bf{x}}}_{N}(t)\}).$$

Clearly there exist simple policies in ${\Upsilon }_{\infty }$ under which *g*_*σ*_ will be a finite number. We are interested in the optimal one that minimizes *g*_*σ*_.

Recall the definition of **Q**(*u*, *y*). Similarly, the optimal policy σ* that minimizes *g*_*σ*_ is Markovian. In fact, it is also stationary in the sense that ${\sigma }_{t}^{\ast }({\bf{x}}(t)=x)={\sigma }_{s}^{\ast }({\bf{x}}(s)=x)$ for all *s*, *t* ≥ 0. Define cost-to-go function

$$G(x):=\mathop{\min }\limits_{\sigma \in {\Upsilon }_{\infty }}{{\mathbb{E}}}_{\sigma }(\mathop{\inf }\limits_{s}\{s\ge \mathrm{0:}\,{{\bf{x}}}_{1}(s+t)=\ldots ={{\bf{x}}}_{N}(s+t)\}|{\bf{x}}(t)=x)\mathrm{.}$$

In this case the function *G*(*x*) satisfies the following equation^22^

3 $$G(x)=1+\mathop{{\rm{\min }}}\limits_{u\in { {\mathcal M} }^{N}}\,\sum _{y\in {\{ \ltimes ,\rtimes \}}^{N}}\,{\mathbb{P}}({\bf{y}}(t)=y|{\bf{x}}(t)=x,{\bf{u}}(t)=u)G({\bf{Q}}(u,y)),$$

and the optimal decision σ* is given by

4 $${\sigma }_{t}^{\ast }({\bf{x}}(t)=x)={\rm{\arg }}\,\mathop{{\rm{\min }}}\limits_{u\in { {\mathcal M} }^{N}}\,\sum _{y\in {\{ \ltimes ,\rtimes \}}^{N}}\,{\mathbb{P}}({\bf{y}}(t)=y|{\bf{x}}(t)=x,{\bf{u}}(t)=u)G({\bf{Q}}(u,y\mathrm{)).}$$

#### Computation/Communication Complexities

We would like to point out that the derived optimal network-level rules are conceptually equivalent to the single qubit framework^17^. Although the centralized optimal solutions are clear in theory for both finite and infinite time horizons, it is important to understand the amount of computation and communication resources required for implementing them in practice for a considerably large network.

The Bellman equations (1) and (3) involve a continuous action set ${ {\mathcal M} }^{N}$. Usually this is approximated by a proper discretization of ${\mathcal M}$ into a finite set. For example, we can let the measurements be selected from ref. 18

5 $${{\rm{M}}}_{\alpha }:\alpha =\frac{j\pi }{2K},\,j=\mathrm{0,}\ldots ,K-1.$$

Suppose ${\mathcal M}$ has been discretized into a finite set with *K* elements. Note that this means that the state space ${\mathscr{S}}$ for each qubit is also discretized with 2*K* elements. We now discuss the finite horizon case in detail. From the computational side, solving the Bellman equation (1) relies on recursively along the equation (1) computing *C*(*x*, *t*) (and therefore obtain the optimal ${\sigma }_{t}^{\ast }(x)$) from *C*(*x*, *t* + 1) for all $t=T-1,\ldots ,0$ and all $x\in {{\mathscr{S}}}^{N}$, starting with the boundary condition

$$C(x,T)=\sum _{i,j=1}^{N}\,|\langle {x}_{i}|{x}_{j}\rangle |=\sum _{i,j=1}^{N}\,{|\cos ({x}_{i}-{x}_{j})|}^{2}\mathrm{.}$$

The number of algebraic operations required in such process inevitably grows faster than *K* ^*N*^. Therefore, practically it is almost impossible to numerically solve the Bellman equation (1) and obtain the optimal policy for a large network. In fact, even if the computation can be done off line, preserving the optimal policy relies on $O\mathrm{((2}K{)}^{N}T\,\mathrm{log}\,K)$ bits of memory. Since each node relies on the states of all other nodes to carry out the optimal policy, the network requires all-to-all communications with *O*(*N* ^2^) bits of transmissions per step.

### Distributed Solution

In this section, we discuss distributed solutions to the considered qubit consensus problem in the sense that nodes communicate with a few neighbours locally and then make measurement selection decisions individually.

#### The Algorithm

We assume that there is a connected underlying graph G = (V, E) with node set V and edge set E representing the classical communication links among the nodes, where a link $\{i,j\}\in {\rm{E}}$ specifies that nodes *i* and *j* can exchange information their states. We denote ${{\rm{N}}}_{i}:=\{j:\{i,j\}\in {\rm{E}}\}$ as the neighbour set of node *i*. We also define

$$a\,{\rm{mod}}\,\,\pi \mathrm{/2}=(\begin{array}{ll}a & {\rm{if}}\,a\in \mathrm{[0},\pi \mathrm{/2),}\\ a-\pi \mathrm{/2} & {\rm{if}}\,a\in [\pi \mathrm{/2},\pi )\end{array}$$

and

$$a\,{\rm{mod}}\,\pi =(\begin{array}{ll}a & {\rm{if}}\,a\in \mathrm{[0,}\pi ),\\ a-\pi  & {\rm{if}}\,a\in [\pi \mathrm{,2}\pi \mathrm{).}\end{array}$$

We propose the following algorithm.

**Pairwise Qubit Projection** (**PQP**). (i) At each *t*, a node *i* is drawn uniformly at random from the set V, and then node *j* is selected uniformly at random from the set N_*i*_; (ii) The selected pair of nodes *i* and *j* exchanges their current states **x**_*i*_(*t*) and **x**_*j*_(*t*); (iii) Nodes *i* and *j* apply projective measurements

$${{\bf{u}}}_{i}(t)={{\bf{u}}}_{j}(t)=({{\bf{x}}}_{i}(t)+{{\bf{x}}}_{j}(t\mathrm{))/2}\,{\rm{mod}}\,\pi \mathrm{/2}$$

and all other nodes keep their current states.

We remark that the above algorithm is clearly inspired by the class of gossiping algorithms for classical communication networks and open quantum networks^6, 10, 23^. This proposed algorithm can be realized in fully distributed manner in the sense that nodes even need not to share a common clock and can simply follow independent Poisson processes to wake up^6^. Moreover, the pair section process can also be made deterministic and multiple disjoint pairs can be selected at a given time, which will not change the nature of the algorithm and actually can speed up the algorithm. The involved projective measurements introduce new type of randomness in the algorithm, which makes the PQP algorithm differ from the previous algorithms^6, 10, 23^ at a fundamental level.

#### Agreement Convergence

Let **x**(*t*) be driven by the proposed PQP algorithm. Suppose node pair $\{i,j\}$ is selected at time *t*. Then from the quantum measurement postulate, independently among $m\in \{i,j\}$ we have

$${{\bf{x}}}_{m}(t+\mathrm{1)}=\frac{{{\bf{x}}}_{i}(t)+{{\bf{x}}}_{j}(t)}{2}$$

with probability ${\cos }^{2}\,\frac{{{\bf{x}}}_{i}(t)-{{\bf{x}}}_{j}(t)}{2}$, and

$${{\bf{x}}}_{m}(t+\mathrm{1)}=\frac{{{\bf{x}}}_{i}(t)+{{\bf{x}}}_{j}(t)+\pi }{2}\,{\rm{mod}}\,\pi $$

with probability ${\sin }^{2}\,\frac{{{\bf{x}}}_{i}(t)-{{\bf{x}}}_{j}(t)}{2}$. The following result holds.

**Theorem 1** *Using the PQP algorithm*, *the hybrid quantum network reaches an agreement almost surely in the sense that*

$${\mathbb{P}}(\mathop{\mathrm{lim}}\limits_{t\to \infty }|{{\bf{x}}}_{i}(t)-{{\bf{x}}}_{j}(t)|=0)=1$$

*for all i*, $$j\in {\rm{V}}$$.

**Figure 2:**
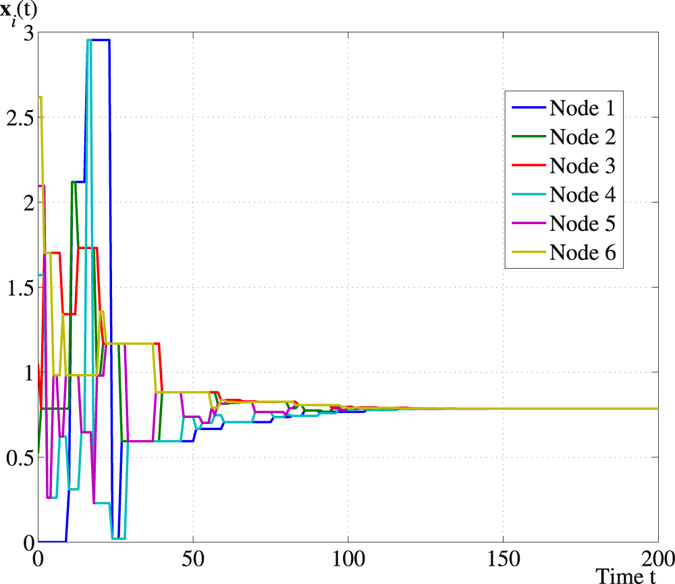
A sample path of **x**_*i*_(*t*), $i=1,\ldots ,6$ along the PQP algorithm with initial value ${{\bf{x}}}_{i}\mathrm{(0)}=(i-1)\pi \mathrm{/6}$, $i=1,\ldots \mathrm{,\; 6}$.

**Figure 3:**
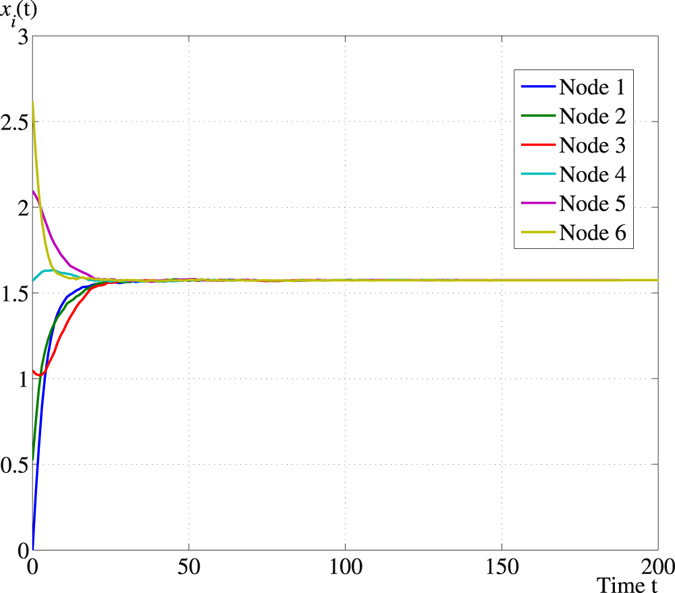
Trajectories of ${x}_{i}(t):={\mathbb{E}}({{\bf{x}}}_{i}(t))$, $i=1,\ldots \mathrm{,\; 6}$ along the PQP algorithm with initial value ${{\bf{x}}}_{i}\mathrm{(0)}=(i-\mathrm{1)}\pi \mathrm{/6}$, $i=1,\ldots \mathrm{,\; 6}$.

For the hybrid quantum network illustrated in Fig. 1, we plot a sample path at which consensus is reached and the trajectories of the expected states of the qubits, respectively, in Figs 2 and 3.

#### The Agreement Limit in Expectation

We introduce ***ρ***_*i*_(*t*) as the density operator corresponding to ${{\bf{x}}}_{i}(t)\in {\mathscr{S}}$. Viewing also **x**_*i*_(*t*) as its angle in [0, *π*), we can formally write:

6 $${{\boldsymbol{\rho }}}_{i}(t)=[\begin{array}{cc}{\cos }^{2}\,{{\bf{x}}}_{i}(t) & \cos \,{{\bf{x}}}_{i}(t)\,\sin \,{{\bf{x}}}_{i}(t)\\ \cos \,{{\bf{x}}}_{i}(t)\,\sin \,{{\bf{x}}}_{i}(t) & {\sin }^{2}\,{{\bf{x}}}_{i}(t)\end{array}]$$

We also define ${\rho }_{i}(t)={\mathbb{E}}\{{{\boldsymbol{\rho }}}_{i}(t)\}$, where ${\mathbb{E}}$ is subject to the classical measure ${\mathbb{P}}$ capturing all randomness in the node pair selection process and in the quantum projective measurements. All the outcomes of the projective measurements have to be read out for carrying out the algorithm. Nonetheless ***ρ***_*i*_(*t*) describes the distribution of ***ρ***_*i*_(*t*) under the measure ${\mathbb{P}}$. We stack ${\boldsymbol{\rho }}(t)={({{\boldsymbol{\rho }}}_{1}(t)\ldots {{\boldsymbol{\rho }}}_{N}(t))}^{{\rm{{\rm T}}}}$ and $\rho (t)={({\rho }_{1}(t)\ldots {\rho }_{N}(t))}^{{\rm{{\rm T}}}}$ as vectors of 2 × 2 density operators.

It turned out that it is more convenient to investigate the evolution of the **x**(*t*) from the corresponding density operators, whose original update is in fact rather complex. Let *L*_G_ be the Laplacian of the graph G, defined by ${[{L}_{{\rm{G}}}]}_{ij}=-\mathrm{(1/}|{{\rm{N}}}_{i}|+\mathrm{1/}{|N}_{j}|)$ for $\{i,j\}\in {\rm{E}}$, [*L*_G_]_*ij*_ = 0 for $i\ne j$ with $\{i,j\}\notin {\rm{E}}$, and ${[{L}_{{\rm{G}}}]}_{ii}={\sum }_{j=1}^{N}\,{[{L}_{{\rm{G}}}]}_{ij}$. We have the following theorem.

**Theorem 2** *The density vector sequence* ${(\rho (t))}_{t\ge 0}$ *satisfies*

$$\rho (t+\mathrm{1)}=({I}_{N}-{L}_{{\rm{G}}})\otimes {I}_{2}\rho (t),\,t\ge 0.$$

*Consequently*, *we have*

$$\mathop{\mathrm{lim}}\limits_{t\to \infty }\,{\rho }_{i}(t)=\frac{{\sum }_{i=1}^{N}\,{\rho }_{i}\mathrm{(0)}}{N},\,i\in {\rm{V}}$$

*with an exponential rate at* 1 − *λ*_2_(*L*_G_), *where λ*_2_(*L*_G_) *is the smallest positive eigenvalue of L*_G_.

**Figure 4:**
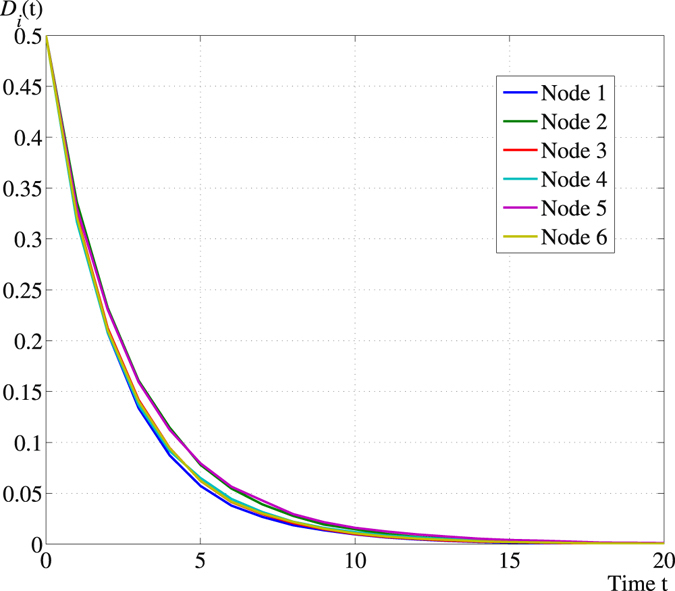
Trajectories of ${D}_{i}(t),\,i=1,\ldots \mathrm{,\; 6}$ along the PQP algorithm with initial value ${{\bf{x}}}_{i}\mathrm{(0)}=(i-\mathrm{1)}\pi \mathrm{/6}$, $i=1,\ldots \mathrm{,\; 6}$.

Theorem 2 shows that in the operator space, ${\mathbb{E}}\{{\boldsymbol{\rho }}(t)\}$ simply follows a linear time-invariant system and eventually leads to an average consensus. This result is related but also in contrast to the work of refs 10 and 11, which showed that a network of qubits interconnected by local environments can be driven to a consensus of their individual reduced states. The evolution of ${\mathbb{E}}\{{\bf{x}}(t)\}$ is on the other hand highly complex for which even a nonlinear recursive form is out of reach. An illustration of Theorem 2 is presented below for the hybrid quantum network in Fig. 4, where we plot

$${D}_{i}(t):={\Vert {\rho }_{i}(t)-\frac{{\sum }_{i=1}^{N}{\rho }_{i}\mathrm{(0)}}{N}\Vert }^{2}$$

with ||·|| being the matrix 2-norm for $i=1,\ldots ,6$, respectively.

## Discussions

We should emphasize that although both the centralized and distributed solutions are able to drive the quantum network to certain agreement states, the respective agreement limits have different indications. Due to the feedback nature of the centralized scheme, from the resulting agreement state limited information can be recovered on the network initial values. In this sense the quantum agreement under centralized decisions shares very little spirit in common with consensus seeking over classical networks^3, 6^. The usefulness of such a framework then lies in how we have resolved a global optimization problem over quantum networks with the help of classical communication and computing, shedding light on other optimal operation problems arising from quantum information processing, e.g., source allocation and packet routing. On the other hand, the presented distributed solution provides an alternative way of realizing quantum average consensus at least in the sense of expected network state, besides the open quantum network approach^10, 11^. On top of that more sophisticated protocols can be naturally expected, potentially leading to useful quantum algorithms as we have witnessed the parallel development for classical complex networks^1, 2^.

It is also worth noting that there are no fundamental obstacles in generalizing these results to more complex hybrid quantum networks. When coherent quantum states and joint measurements are involved, feasible network states and control actions will yield larger state spaces. However, the outcomes of the local quantum operations continue to depend only on the current state and control decisions at each individual node. Therefore, the resulting centralized schemes will define similar stochastic optimal control problems, which can be solved using the same methodologies. Decentralized schemes in that case have to rely on new algorithms that take into consideration features of the new state space and transition behaviors, but the critical insight remains to be using simple diffusive node interactions to create collective network state convergence.

## Methods

Note that ***ρ***_*i*_(*t*) is a random variable, whose realizations are always pure states. The expected value *ρ*_*i*_(*t*) of ***ρ***_*i*_(*t*), is in general a mixed state.

### Proof of Theorem 1

Define ${p}_{ij}=\mathrm{(1/}|{{\rm{N}}}_{i}|+\mathrm{1/}{|N}_{j}|)$ for $\{i,j\}\in {\rm{E}}$ as the probability of link {*i*, *j*} being selected at a given time. Introduce ${\bf{h}}(t):={\sum }_{\{i,j\}:i < j}\,{\rm{Tr}}({{\boldsymbol{\rho }}}_{i}(t){{\boldsymbol{\rho }}}_{j}(t))$. Then we have

7 $$\begin{array}{l}{\mathbb{E}}({\bf{h}}(t+\mathrm{1)|}{\boldsymbol{\rho }}(t))\\ \begin{array}{rcl} & = & \sum _{\{k,m\}\in {\rm{E}}}\,{p}_{km}{{\mathbb{E}}}_{km}({\bf{h}}(t+\mathrm{1)|}{\boldsymbol{\rho }}(t))\\  & = & \sum _{\{k,m\}\in {\rm{E}}}\,{p}_{km}{{\mathbb{E}}}_{km}[{\rm{Tr}}({{\boldsymbol{\rho }}}_{k}(t+\mathrm{1)}{{\boldsymbol{\rho }}}_{m}(t+\mathrm{1))}\\  &  & +\sum _{\{i,j\}:i < j,\{i,j\}\ne \{k,m\}}\,{\rm{Tr}}({{\boldsymbol{\rho }}}_{i}(t+\mathrm{1)}{{\boldsymbol{\rho }}}_{j}(t+\mathrm{1))|}{\boldsymbol{\rho }}(t)]\\  & = & \sum _{\{k,m\}\in {\rm{E}}}\,{p}_{km}({\rm{Tr}}[{{\mathbb{E}}}_{km}({{\boldsymbol{\rho }}}_{k}(t+\mathrm{1)}{{\boldsymbol{\rho }}}_{m}(t+\mathrm{1))|}{\boldsymbol{\rho }}(t)]\\  &  & +{\rm{Tr}}[\sum _{\{i,j\}:i < j,\{i,j\}\ne \{k,m\}}\,{{\mathbb{E}}}_{km}({{\boldsymbol{\rho }}}_{i}(t+\mathrm{1)}{{\boldsymbol{\rho }}}_{j}(t+\mathrm{1))|}{\boldsymbol{\rho }}(t)])\end{array}\end{array}$$

where in the first equality the ${{\mathbb{E}}}_{km}$ is subject to the randomness generated by quantum measurements at nodes *k* and *m*, and in the last equality we have used the fact that trace and expectation commute due to their linearity.

Proceeding with the first of the two trace terms in the right-hand side of (7), we have

8 $$\begin{array}{l}{\rm{Tr}}[{{\mathbb{E}}}_{km}({{\boldsymbol{\rho }}}_{k}(t+\mathrm{1)}{{\boldsymbol{\rho }}}_{m}(t+\mathrm{1))|}{\boldsymbol{\rho }}(t)]\\ \begin{array}{rcl} & = & {\rm{Tr}}[{{\mathbb{E}}}_{km}({{\boldsymbol{\rho }}}_{k}(t+\mathrm{1))}{{\mathbb{E}}}_{km}({{\boldsymbol{\rho }}}_{m}(t+\mathrm{1))|}{\boldsymbol{\rho }}(t)]\\  & = & {\rm{Tr}}(\frac{1}{2}{{\boldsymbol{\rho }}}_{k}(t)+\frac{1}{2}{{\boldsymbol{\rho }}}_{m}(t))\,(\frac{1}{2}{{\boldsymbol{\rho }}}_{k}(t)+\frac{1}{2}{{\boldsymbol{\rho }}}_{m}(t))\\  & = & \frac{1}{2}+\frac{1}{2}{\rm{Tr}}({{\boldsymbol{\rho }}}_{k}(t){{\boldsymbol{\rho }}}_{m}(t)),\end{array}\end{array}$$

where the first equality is due to independence of the outcome of the quantum measurements at nodes *k* and *m*, and the second equality utilizes (12). Meanwhile, considering the second trace term in the right-hand side of (7), it is easy to conclude from (12) that

9 $$\begin{array}{c}{\rm{Tr}}[\sum _{\{i,j\}:i < j,\{i,j\}\ne \{k,m\}}\,{{\mathbb{E}}}_{km}({{\boldsymbol{\rho }}}_{i}(t+\mathrm{1)}{{\boldsymbol{\rho }}}_{j}(t+\mathrm{1))|}{\boldsymbol{\rho }}(t)]\\ \quad =\sum _{\{i,j\}:i < j,\{i,j\}\ne \{k,m\}}\,{\rm{Tr}}({{\boldsymbol{\rho }}}_{i}(t){{\boldsymbol{\rho }}}_{j}(t\mathrm{)).}\end{array}$$

As a result, from (7), (8) and (9) we have

10 $$\begin{array}{l}{\mathbb{E}}({\bf{h}}(t+\mathrm{1)|}{\boldsymbol{\rho }}(t))\\ \quad =\sum _{\{k,m\}\in {\rm{E}}}{p}_{km}[{\bf{h}}(t)+\tfrac{1}{2}\mathrm{(1}-{\rm{T}}r({{\boldsymbol{\rho }}}_{k}(t){{\boldsymbol{\rho }}}_{m}(t)))]\\ \quad ={\bf{h}}({\bf{t}})+\sum _{\{k,m\}\in {\rm{E}}}\,\frac{{p}_{km}}{2}\mathrm{(1}-{\rm{Tr}}({{\boldsymbol{\rho }}}_{k}(t){{\boldsymbol{\rho }}}_{m}(t)))\end{array}$$

Since ${\rm{Tr}}({{\boldsymbol{\rho }}}_{k}(t){{\boldsymbol{\rho }}}_{m}(t))\le 1$ always holds, (10) implies that {**h**(*t*)} is a submartingale. Moreover, ${\mathbb{E}}({\bf{h}}(t))\le N(N-\mathrm{1)/2}$ for all *t* by the definition of **h**(*t*). By the Martingale Convergence Theorem (Theorem 5.2.8^24^), **h**(*t*) converges to a finite limit almost surely. We can further invoke the Dominated Convergence Theorem (e.g., Exercise 2.3.7^24^) to yield that, ${\mathbb{E}}({\bf{h}}(t))$ converges to a finite limit. Hence, (10) implies

$${\mathbb{E}}({\bf{h}}(t+\mathrm{1))}={\mathbb{E}}({\bf{h}}(t))+{\mathbb{E}}[\sum _{\{k,m\}\in {\rm{E}}}\,\frac{{p}_{km}}{2}\mathrm{(1}-{\rm{Tr}}({{\boldsymbol{\rho }}}_{k}(t){{\boldsymbol{\rho }}}_{m}(t)))],$$

so ${\mathrm{lim}}_{t\to \infty }\,{\mathbb{E}}({\rm{Tr}}({{\boldsymbol{\rho }}}_{k}(t){{\boldsymbol{\rho }}}_{m}(t)))=1$ for all $\{k,m\}\in {\rm{E}}$. However, as ${\rm{Tr}}({{\boldsymbol{\rho }}}_{k}(t){{\boldsymbol{\rho }}}_{m}(t))\le 1$ is a sure event, we conclude for any $\varepsilon  > 0$ that

11 $$\mathop{\mathrm{lim}}\limits_{t\to \infty }\,{\mathbb{P}}({\rm{Tr}}({{\boldsymbol{\rho }}}_{k}(t){{\boldsymbol{\rho }}}_{m}(t))\ge 1-\varepsilon )=1,$$

i.e., ${\rm{Tr}}({{\boldsymbol{\rho }}}_{k}(t){{\boldsymbol{\rho }}}_{m}(t))$ converges to 1 in probability for all $\{k,m\}\in {\rm{E}}$.

Finally, we notice that ${\rm{Tr}}({{\boldsymbol{\rho }}}_{k}(t){{\boldsymbol{\rho }}}_{m}(t))={\cos }^{2}\,({{\bf{x}}}_{k}(t)-{{\bf{x}}}_{m}(t))$. Therefore, ${\rm{Tr}}({{\boldsymbol{\rho }}}_{k}(t){{\boldsymbol{\rho }}}_{m}(t))$ converging to one in probability is equivalent to that **x**_*k*_(*t*) − **x**_*m*_(*t*) converging to zero in probability. While G is a connected graph, we further know that **x**_*i*_(*t*) − **x**_*j*_(*t*) converges to zero in probability for all *i*, $j\in {\rm{V}}$. This immediately implies that **h**(*t*) will converge to *N*(*N* − 1)/2 in probability. However, we have known as a fact that **h**(*t*) converges in the almost sure sense. Therefore, **h**(*t*) must converge to *N*(*N* − 1)/2 almost surely, or equivalently, **x**_*i*_(*t*) − **x**_*j*_(*t*) converging to zero almost surely for all *i*, $j\in {\rm{V}}$. The desired theorem holds and we have now completed the proof.

### Proof of Theorem 2

Suppose node pair $\{i,j\}$ is selected at time *t*. Then based on (6), we obtain

12 $$\begin{array}{l}{\mathbb{E}}\{{{\boldsymbol{\rho }}}_{i}(t+\mathrm{1)|}{\boldsymbol{\rho }}(t)\}\\ \begin{array}{rcl} & = & {\mathbb{E}}\{{{\boldsymbol{\rho }}}_{j}(t+\mathrm{1)|}{\boldsymbol{\rho }}(t)\}\\  & = & {\cos }^{2}\,\tfrac{{{\bf{x}}}_{i}(t)-{{\bf{x}}}_{j}(t)}{2}[\begin{array}{cc}{\cos }^{2}\,\tfrac{{{\bf{x}}}_{i}(t)+{{\bf{x}}}_{j}(t)}{2} & \cos \,\tfrac{{{\bf{x}}}_{i}(t)+{{\bf{x}}}_{j}(t)}{2}\,\sin \,\tfrac{{{\bf{x}}}_{i}(t)+{{\bf{x}}}_{j}(t)}{2}\\ \cos \,\tfrac{{{\bf{x}}}_{i}(t)+{{\bf{x}}}_{j}(t)}{2}\,\sin \,\tfrac{{{\bf{x}}}_{i}(t)+{{\bf{x}}}_{j}(t)}{2} & {\sin }^{2}\,\tfrac{{{\bf{x}}}_{i}(t)+{{\bf{x}}}_{j}(t)}{2}\end{array}]\\  &  & +{\sin }^{2}\,\tfrac{{{\bf{x}}}_{i}(t)-{{\bf{x}}}_{j}(t)}{2}[\begin{array}{cc}{\sin }^{2}\,\tfrac{{{\bf{x}}}_{i}(t)+{{\bf{x}}}_{j}(t)}{2} & -\,\cos \,\tfrac{{{\bf{x}}}_{i}(t)+{{\bf{x}}}_{j}(t)}{2}\,\sin \,\tfrac{{{\bf{x}}}_{i}(t)+{{\bf{x}}}_{j}(t)}{2}\\ -\,\cos \,\tfrac{{{\bf{x}}}_{i}(t)+{{\bf{x}}}_{j}(t)}{2}\,\sin \,\tfrac{{{\bf{x}}}_{i}(t)+{{\bf{x}}}_{j}(t)}{2} & {\cos }^{2}\,\tfrac{{{\bf{x}}}_{i}(t)+{{\bf{x}}}_{j}(t)}{2}\end{array}]\\  & = & \tfrac{1}{2}[\begin{array}{cc}{\cos }^{2}\,{{\bf{x}}}_{i}(t) & \cos \,{{\bf{x}}}_{i}(t)\,\sin \,{{\bf{x}}}_{i}(t)\\ \cos \,{{\bf{x}}}_{i}(t)\,\sin \,{{\bf{x}}}_{i}(t) & {\sin }^{2}\,{{\bf{x}}}_{i}(t)\end{array}]\\  &  & +\tfrac{1}{2}[\begin{array}{cc}{\cos }^{2}\,{{\bf{x}}}_{j}(t) & \cos \,{{\bf{x}}}_{j}(t)\,\sin \,{{\bf{x}}}_{j}(t)\\ \cos \,{{\bf{x}}}_{j}(t)\,\sin \,{{\bf{x}}}_{j}(t) & {\sin }^{2}\,{{\bf{x}}}_{j}(t)\end{array}]\\  & = & \tfrac{1}{2}{{\boldsymbol{\rho }}}_{i}(t)+\tfrac{1}{2}{{\boldsymbol{\rho }}}_{j}(t),\end{array}\end{array}$$

where the third equality holds from elementary sum-to-product trigonometric formulas. We further obtain

$$\rho (t+\mathrm{1)}=({I}_{N}-{L}_{{\rm{G}}})\otimes {I}_{2}\rho (t).$$

by collecting all events at the pairs of nodes. The convergence statement aligns with the same argument as used in ref. 6. This concludes the proof.

## Conclusions

We have considered a consensus seeking problem over a quantum hybrid network. A number of nodes each holding a qubit apply projective measurements and the measurement results are exchanged via classical communications. Centralized optimal path planning for the network with all-to-all classical communications were derived by stochastic optimal control approach, whose overwhelming computation and communication complexities were shown for a large network. A distributed Pairwise Qubit Projection (PQP) algorithm was also proposed along which the qubit states can be driven to a consensus almost surely along the proposed PQP algorithm. Future work includes generalization of the optimal control and distributed control approaches to hybrid quantum networks in the presence of quantum links as entangled pairs for improving the efficiency and scalability of such networks in applications.
